# Anticoagulation Control in Different Ethnic Groups Receiving Vitamin K Antagonist for Stroke Prevention in Atrial Fibrillation

**DOI:** 10.3389/fcvm.2021.736143

**Published:** 2021-11-16

**Authors:** Nur Azyyati Zawawi, Izzati Abdul Halim Zaki, Long Chiau Ming, Hui Poh Goh, Hanis Hanum Zulkifly

**Affiliations:** ^1^Department of Pharmacy Practice, Fakulti Farmasi, Universiti Teknologi MARA (UiTM), Bandar Puncak Alam, Malaysia; ^2^Cardiology Therapeutics Research Group, Universiti Teknologi MARA, Bandar Puncak Alam, Malaysia; ^3^Pengiran Anak Puteri Rashidah Sa'adatul Bolkiah (PAPRSB) Institute of Health Sciences, Universiti Brunei Darussalam, Gadong, Brunei

**Keywords:** atrial fibrillation, ethnicity, time in therapeutic range (TTR), adverse clinical outcomes, anticoagulant

## Abstract

Vitamin K antagonist such as warfarin reduces the risk of stroke in atrial fibrillation (AF) patients. Since warfarin has a narrow therapeutic index, its administration needs to be regularly monitored to avoid any adverse clinical outcomes such as stroke and bleeding. The quality of anticoagulation control with warfarin therapy can be measured by using time in therapeutic range (TTR). This review focuses on the prevalence of AF, quality of anticoagulation control (TTR) and adverse clinical outcome in AF patients within different ethnic groups receiving warfarin therapy for stroke prevention. A literature search was conducted in Embase and PubMed using keywords of “prevalence,” “atrial fibrillation,” “stroke prevention,” “oral anticoagulants,” “warfarin,” “ethnicities,” “race” “time in therapeutic range,” “adverse clinical outcome,” “stroke, bleeding.” Articles published by 1st February 2020 were included. Forty-one studies were included in the final review consisting of AF prevalence (*n* = 14 studies), time in therapeutic range (*n* = 18 studies), adverse clinical outcome (*n* = 9 studies) within different ethnic groups. Findings indicate that higher prevalence of AF but better anticoagulation control among the Whites as compared to other ethnicities. Of note, non-whites had higher risk of strokes and bleeding outcomes while on warfarin therapy. Addressing disparities in prevention and healthcare resource allocation could potentially improve AF-related outcomes in minorities.

## Introduction

Atrial fibrillation (AF) is an atrial tachyarrhythmia that has an uncoordinated atrial activation, with consequent atrial mechanical function deterioration (1). Based on the latest European Society of Cardiology Guidelines, AF had remained one of the world's main causes of stroke, heart failure, sudden death and cardiovascular disease (2). The prevalence of AF has been increasing throughout the world affecting 8 million people in Europe and this is expected to increase 2-3-fold by 2030 (3). Older age, male sex, diabetes, and ischemic heart disease are factors associated with AF diagnosis (4). The use of Vitamin K antagonist (VKA) in AF patients is associated with a reduction in the risk of thromboembolic complications when time in therapeutic range (TTR) >70% is achieved (5). TTR is the time spent within the therapeutic range of INR (2.0–3.0) (6). Patients with TTR ≥70% are considered to have a well-controlled warfarin therapy while those with TTR ≤ 70% are considered as poorly controlled warfarin therapy (6). Current research suggests that high TTR in patients treated with warfarin for AF correlates with better patient outcomes (6). However, challenges arise in identifying patients who are likely to achieve and maintain therapeutic INR as well as good anticoagulation control. The quality of anticoagulation control can be influenced by many factors. Ethnicity has been established as one of the factors that might impact anticoagulation control (7). A clinical scoring system, the SAMe-TT_2_R_2_ score was developed in 2013 that presents the most common clinical and demographic factors that might influence anticoagulation control in AF patients (7) and non-white ethnicity is included in this scoring system. The score can be used to aid decision making by identifying those patients who would probably do well when treated with VKA (achieving a high TTR, >65%) or, conversely, those who would need additional interventions to achieve good INR control or to be initiated on/switched to a non-VKA oral anticoagulant (NOAC) (7).

Stroke is a long-term complication related to AF when effective measures of stroke prevention is not taken (2). Stroke tends to be more severe with a higher recurrence in AF patients reaching 6.9% compared to 4.7% in patients with stroke without atrial fibrillation (2). The CHA_2_DS_2_-VASc score, a well-validated score has been used worldwide to identify AF patients' risk factors of stroke and the need for oral anticoagulation therapy. Patients with a low risk factor of stroke from the CHA_2_DS_2_-VASc (CHA_2_DS_2_-VASc 0 in male and 1 in female) do not require antithrombotic treatment, while patients with risk factors for strokes (i.e., CHA_2_DS_2_-VASc of 1 or more for males and 2 or more for females) require oral anticoagulation therapy to prevent the risk of stroke (2). Vitamin K antagonist (e.g., warfarin) is one of the effective oral anticoagulants (OAC) that can be used to reduce the risk of stroke from AF by 64% (8). Nonetheless, bleeding risk is a major concern for AF patients taking warfarin therapy. Uncontrolled monitoring of warfarin therapy is associated with major bleeding in AF patients (9, 10). The risk of bleeding can be measured by using the HAS-BLED scoring system where a risk of bleeding ≥ 3 indicates high-risk bleeding and a score of <3 indicates low risk of bleeding (2).

The prevalence of AF, quality of anticoagulation and adverse clinical outcome among different ethnic groups varies and was seen to be poor among non-white ethnicities. Hence, this is the first review that reports on the prevalence of AF, the quality of anticoagulation control (TTR) and adverse clinical outcome among different ethnic groups receiving warfarin therapy for stroke prevention.

## Method

**Figure 1:**
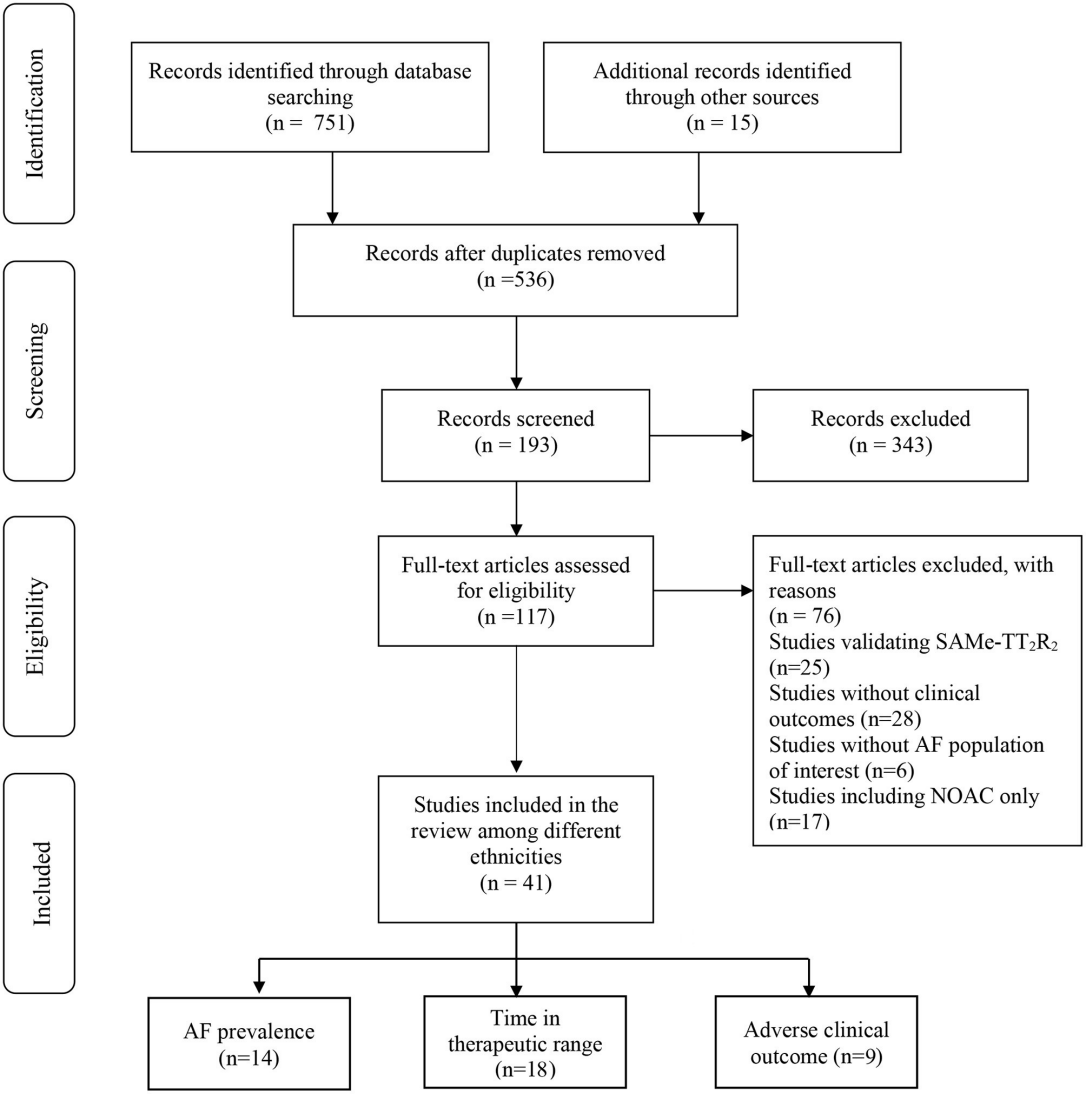
Selection of studies included in the review.

A literature search was conducted on Embase and PubMed using keywords of “prevalence,” “atrial fibrillation,” “stroke prevention,” “oral anticoagulants,” “warfarin,” “ethnicities,” “race” “time in therapeutic range,” “adverse clinical outcome,” “stroke, bleeding.” Articles published by 1st February 2020 were included. Given the lack of large scale RCTs in this population, evidence from prospective and retrospective studies were incorporated. Besides, they serve as “real-world” evidence on oral anticoagulant (OAC) use in AF patients with different ethnicities. Forty-one studies were included in the final review consisting of AF prevalence (*n* = 14 studies), time in therapeutic range (*n* = 18 studies), adverse clinical outcome (*n* = 9 studies) within different ethnic groups (Figure 1).

## AF Among Different Ethnic Groups

The prevalence of AF differs between each ethnics group. Table 1 represents 14 studies (11–24) on the prevalence of AF based on different ethnic groups. According to all studies, White people have the highest prevalence of AF compared to Afro-Caribbean, Asian, Hispanic and others ranging from 1.2% (12) to 29% (19). Only 3 studies (11, 22, 24) investigated AF diagnosis within the Hispanics ranging from 2.6 to 7.8%. While eight other studies (11–13, 15, 18, 20, 22, 24) reported on AF prevalence within Asians ranging from 0.05 to 10.1%. There was only one study (24) reported prevalence of AF among Native American (4.4%) and Pacific Islandar/Hawaiian (4.6%) and another study (15) which reported the prevalence of AF in Malaysia according to its ethnic groups with Malay (0.77%), Chinese (0.05%), others (0.06%). Besides, a study by Shavadia et al. (25) also reported that Asian ethnicity has been associated with considerably lower AF rates compared to White ethnicity. Differences in clinically detected AF among different ethnic group might be evident. It could reflect the variations in clinical recognition of AF, perception of AF symptoms or access to health care, the limited participation of minorities in trials and clinical studies for AF; or also due to difference in the completeness of clinical assessment when patients are presented with AF symptoms (11).

**Table 1:** Prevalence of AF by ethnicity.

**Country**	**References**	**a) Study design**	**Prevalence (%)**				
		**b) Follow-up**					
		**c) Sample size**	**White**	**Black**	**Asian**	**Hispanic**	**Others**
US	Heckbert et al. (11)	a) Cross sectional	11.3	6.6	9.9 - Chinese	7.8	-
		b) 14.4 years					
		c) 1,556					
London	Mathur et al. (12)	a) Cross sectional	1.2	0.4	0.2 - South	-	-
		b) 3 years			Asian		
		c) 6,292					
England	Gillot et al. (13)	a) Observational	2.4	-	0.4 - South	-	-
		b) N/A			Asian		
		c) 277,218					
US	Magnani et al. (14)	a) Prospective	8.1	5.8	-	-	-
		b) 6.2 years					
		c) 15,080					
Malaysia	Lim et al. (15)	a) Prospective	-	-	0.77 - Malay	-	
		b) 3 years			0.05- Chinese		
		c) 10,805			0.06 - Other		
California, Florida, New York	Kamel et al. (16)	a) Retrospective	25.5	21.4	-	-	-
		b) 4 years					
		c) 101,773					
Washington	Jensen et al. (17)	a) Prospective	19	17	-	-	-
		b) 11.2 years					
		c) 1,585					
North America, Europe, Asia	Lau et al. (18)	a) Prospective	18	8.3	10.1- Chinese	-	-
		b) 2.5 years			9.5- Japanese		
		c) 2,580					
US	Lahiri et al. (19)	a) Retrospective	29	19	-	-	-
		b) 4 years					
		c) 1,001					
US	Winkelmayer et al. (20)	a) Cross sectional	14	6.5	9.0- South	-	-
		b) 15 years			Asian		
		c) 2,483,199					
US, Washington	Marcus et al. (21)	a) Combination CHS and ARIC study	23	15	-	-	-
		b) N/A					
		c) 19,784					
California	Shen et al. (22)	a) Cross sectional	8	3.8	3.9 - East	3.6	-
		b) 1 year			Asian		
		c) 430,317					
California	Go et al. (23)	a) Cross sectional	2.2	1.5	-	-	-
		b) 1 year					
		c) 17,974					
US	Borzecki et al. (24)	a) Cross sectional	6.1	2.6	3.4	2.6	4.4- Native
		b) 1 year					American
		c) 664,654					4.6- Pacific
							Islandar/Hawaiian

## Time in Therapeutic Range Among Warfarin Users Within Different Ethnic Groups

Time within the therapeutic range (TTR) is used to evaluate anticoagulation control in patients on warfarin therapy for stroke prevention in AF. TTR has a significant impact on patient outcomes such as stroke and mortality (6, 26). As shown in Table 2, most of the studies used TTR ≥ 70 as their cut-off point indicating good anticoagulant control in AF patients. Moreover, there were also other studies (30, 32) that used TTR ≥ 60 and TTR ≥ 65 as their cut-off point to indicate good anticoagulant control among their AF patients.

**Table 2:** Mean TTR of patients among different ethnic group.

**Country**	**References**	**a**.	**Study design**	**a**.	**Sample size**	**Method INR monitoring**	**Mean TTR**
		**b**.	**length of follow up**	**b**.	**race/ethnicity**		
United Kingdom	Zulkifly et al. (27)	a.	Retrospective	a.	1,070	Anticoagulant	White = 67.9
		b.	11 months	b.	Whites, Afro Caribbean, South Asian	clinic	Afro- Caribbean = 61.3
							South Asian = 60.5
Australia	Nguyen et al. (28)	a.	Retrospective	a.	512	Hospital based	Indigenous = 40 (29)
		b.	n/a	b.	Indigenous, non-Indigenous		non-Indigenous = 50 (31)
China	Li et al. (29)	a.	Prospective	a.	379	Hospital based	Overall mean
		b.	3 months	b.	Chinese		TTR 58.35 (26.3)
Lithuania	Urbonas et al. (30)	a.	Retrospective	a.	406	Primary health care centre	TTR > 65 = 20.4
		b.	12 months	b.	-		TTR <65 = 79.6
Spain	Roldán Rabadán et al. (31)	a.	Prospective	a.	1,584	Hospital based	TTR > 70 = 40
		b.	3 years	b.	Whites		TTR <70 = 60
Australia	Bernaitis et al. (9)	a.	Retrospective	a.	3,199	Hospital based	TTR > 70 = 82
		b.	6 months	b.	Whites		TTR <70 = 20
							
Qatar	Mohammed et al. (32)	a.	Retrospective	a.	241	Anticoagulant clinic	TTR > 65 = 65.1
		b.	>6 months	b.	Asian		TTR <65 = 34.9
							Mean TTR:
							Arab = 71
							Asian= 67
Singapore	Bernaitis et al. (33)	a.	Retrospective	a.	1,137	Hospital based	Mean TTR:
		b.	6 months	b.	Malay		Malay = 55.2
					Chinese		Chinese = 58.7
					Indian		Indian = 49.7
Iran	Abbasinazari et al. (34)	a.	Cross sectional	a.	470	Anticoagulant clinic	TTR > 70 = 37.3
		b.	6 months				TTR <70 = 62.7
Malaysia	Yap et al. (35)	a.	Retrospective	a.	500	Hospital based	TTR > 70 = 53.2
		b.	>12 months	b.	Asian		TTR <70 = 46.8
China	Chan et al. (36)	a.	Retrospective	a.	1,428	Hospital based	TTR > 70 = 10.7
		b.	14 years	b.	Chinese		TTR <70 = 89.3
California	Golwala et al. (37)	a.	Prospective	a.	9,542	Outpatient	Mean TTR:
		b.	15 months	b.	White		White = 68
					Black		Black = 59
					Hispanic		Hispanic = 62
North America, Europe, Australia	Lip et al. (38)	a. b.	Randomised controlled trial (RCT) 2 years	a. b.	2,718 White, Black, Asian, Non-Hispanic, Hispanic	RCT	Mean TTR: White; 55.2 Black: 44.0 Asian: 67.0 Non-Hispanic: 53.7 Hispanic: 47.8
US	Yong et al. (39)	a.	Retrospective	a.	184, 161	Outpatient setting	Mean TTR
		b.	1 year	b.	Blacks, whites		White: 57%
							Black: 49%; *p* < 0.001
South Dakota, US	Barta et al. (40)	a. b.	Retrospective 16 months	a. b.	837 Native American Asian Caucasian/white	Tertiary care clinic	TTR > 70 = 43.9 TTR <70 = 56.1
United Kingdom	MacEdo et al. (41)	a.	Population based	a.	29,717	Hospital based	TTR > 70 = 44
		b.	12 months	b.	White		TTR <70 = 56
					Black		
					Asian		
Portugal	Calderia et al. (26)	a. b.	Retrospective >12 months	a. b.	274 Whites	Anticoagulant clinic	TTR > 60 = 53.3 TTR <60 = 46.7
Iran	Singer et al. (42)	a.	Prospective/ Clinical trial	a. b.	1,178 White	Clinical trial center	Mean TTR White = 56.3
					African/American		African/American = 51.9
					Asian		Asian = 48.3
					American		American
					Indian/Alaskan		Indian/Alaskan = 51.2
					Hawaiian		Hawaiian = 52.6

Among the 18 studies (9, 26–42) from the findings, eight studies (27, 28, 32, 33, 37–39, 42) stated TTR among each specific ethnic groups in their population and the other 10 studies (9, 29–31, 34–37, 40, 41) only mentioned the TTR in their overall population (whites, Asians). So far, in Malaysia, there is no study being conducted that focused on TTR among different ethnic groups. However, a study by Yap et al. (35) at Malaysia's National Heart Institute reported only 53.2% of their overall patients had a TTR ≥ 70. Furthermore, in one Singaporean study (33), Chinese patients were reported to have higher mean TTR than Malays with 58.7 and 55.2%, respectively. One study in China (36) showed the percentage of people with TTR≥70 was only 10.7% while TTR ≤ 70% was 89.3% which indicated poor anticoagulation control of warfarin therapy in both countries. Interestingly, Nguyen et al. (28) also reported lower TTR among their 512 Indigenous vs. non-indigenous Australian patients with AF [40 (29) vs. 50 (31); *p* = 0.006]. Based on the previous study by Golwala et al. (37) black individuals had lower median TTRs (59%) than Hispanic (62%) and white (68%) participants; consistent with findings from the sub study of IMPACT trial (38). In relation to a previous study by Zulkifly et al. (27) the researchers also found that the quality of anticoagulant control differs based on ethnicity whereby South Asians and Afro-Caribbeans had poor anticoagulant control with their mean TTR of 60.5 and 61.3%, respectively as opposed to White people with 67.9% (27). Hence, these studies indicate that white people have better anticoagulant control compared to other ethnic groups. Birman-Deych et al. (43) claimed that warfarin did not offer advantages in blacks and Hispanics, partially due to less effective warfarin care and anticoagulation monitoring.

These observations may be due to various reasons, for example differences comorbid disease, socioeconomic status, poor understanding of therapy, adherence issue and genetic background. Ethnic differences in anticoagulation control were evident in a cohort of 9,542 patients (37) receiving warfarin therapy for various indications (AF, VTE, and other mixed conditions), with lower mean TTR among the Blacks compared to Whites. Blacks were younger and lived in areas of highest quartile of poverty, had higher illness burden including more comorbid disease, requiring more medications and hospitalisations to manage those conditions compared to White patients (37). After accounting for all these factors, which are mostly non-modifiable, Black patients still had a recorded TTR 2.3% lower than White patients (37). Meanwhile, poor TTR among Asians might be affected by their dietary intake and extensive use of herbal medications (44). Furthermore, it is not common to have a structured anticoagulant clinic in many parts of the Asian countries causing more challenges in optimising INR control (44).

In terms of pharmacogenetics, warfarin metabolism and dose requirements might differ between ethnic groups. Studies have shown that warfarin dosage requirements are higher in Blacks compared to Whites partly due to racial differences in genotype frequencies (45). Blacks have been found to have additional CYP2C9 alleles which are associated with reduced function of the CYP2C9 activity and thus might contribute to dose variability (45). In addition, issues like variability of health literacy, adherence to medication might also contribute to the differences in quality of anticoagulation therapy among different ethnic groups (37). Perhaps these issues could “flag” the physicians to have a closer and more frequent follow up among ethnic minority patients who are having difficulties in achieving therapeutic INR with warfarin therapy. Otherwise, if without budget constraint, NOACs are in preference to Vitamin K antagonist (VKA) in these patients based on the latest ESC guideline on management of AF (evidence grade 1C) (2).

## Adverse Clinical Outcome Among AF Patients Within Different Ethnic Groups

Warfarin, apart from having a narrow therapeutic index which requires frequent INR monitoring, multiple major drug-drug interaction and drug-food interaction have been documented. Its usage is also associated with adverse event such as thromboembolic and bleeding complications if the quality of anticoagulation control is not optimized. According to Pastori et al. (4) good TTR (>70%) is associated with a low risk of stroke and bleeding. Table 3 outlines adverse clinical outcomes among different ethnic groups based on 9 studies (10, 27, 36, 38, 46–50). Five studies (27, 36, 46–48) focused on the adverse clinical outcomes in each ethnic group while the other four studies (10, 38, 49, 50) focused on the overall population. Referring to study Shen et al. (47). African American or black people has a high risk of stroke compared to other ethnic groups. Similarly, Zulkifly et al. (27) reported that black people had the highest proportion of stroke and bleeding complications (9.8 and 6.5%) compared to White (4.5 and 4.5%) and Asian (4.9 and 5.9%) population, respectively. A meta-analysis of 10 studies comparing the prevalence of AF among African Americans to Whites in the United States concluded that being African American was associated with a “protective effect” from AF [OR 0.51 (95% CI 0.44–0.59); *p* < 0.001]. Despite that, African Americans have twice the risk of first ever stroke compared to Whites and this might be due to higher risk factor burden of stroke, for example, hypertension (51).

**Table 3:** Percentage of adverse clinical outcome by ethnicity.

	**Adverse outcome (%)**						
**References**	**Adverse outcome**	**White**	**Black**	**Asian**	**Hispanic**	**Others**	**Overall population**
Kabra et. al (46)	Stroke	23	38 - African American	-	27.8	28.7 – Native American	-
						22.8– Pacific islander	
Shen et. al (47)	ICH	0.34	0.77	1.75	0.73	-	-
Chan et. al (36)	IS	-	-	23.7 – Chinese	-	-	-
Wang et. al (48)	ICH	-	-	11	-	0.97 – non-Asian	-
Zulkifly et. al (27)	Stroke / TIA	4.5	6.5	4.9	-	-	-
	Bleeding	4.5	9.8	5.9	-	-	-
	CVS hospitalization	21.3	25.6	32.3	-	-	-
	Death	2.5	1.2	2.0	-	-	-
Graham et al. (49)	Bleeding	-	-	-	-	-	1.3
Lip et al. (38)	Bleeding	-	-	-	-	-	1.3–7.4
	ICH	-	-	-	-	-	0.3–2.5
Guo et al. (10)	Bleeding	-	-	-	-	-	1–1.5
	ICH	-	-	-	-	-	0.4
Guo et al. (50)	Major bleeding	-	-	-	-	-	1.14
	Major bleeding + ICH	-	-	-	-	-	0.52

*ICH, intracranial haemorrhage; TIA, transient ischemic attack; IS, ischemic stroke*.

Besides stroke and TIA, patients on warfarin therapy are also at risk of getting intracranial haemorrhage (ICH) with double the risk in Asians relative to the Whites (43). Clinical trials (52, 53) have shown low TTR among Asians compared to non-Asians; however, the rates of major bleeding were significantly higher in Asian patients (53, 54). The reasons behind this are not completely understood but one small Chinese study (*n* = 290) (54) reported the presence of cerebral microbleeds (CMBs) which was associated with numerically higher incidence of ICH among their AF patients on warfarin therapy compared to those without CMBs (3.6 vs. 0.7%, *p* = 0.129).

## Strengths and Limitations of the Review

This is the first review summarising the prevalence, quality of anticoagulation control (TTR), and adverse clinical outcome among AF patients on warfarin therapy within different ethnic groups. Clinicians could understand the underlying factors that influence the treatment outcome among these ethnic groups. Rational prescribing of NOAC and warfarin could be improved by having the snapshot view of AF disease burden and their clinical outcomes in terms of stroke prevention. Nevertheless, the findings derived from this review are limited with caveats such as more than half of the included studies are retrospective in nature, the number of patients included were small and not represented in some parts of the world such as the Middle East and areas other than mainland China and Western Europe.

## Conclusion

In conclusion, this review represents the differences in the prevalence of AF, anticoagulation control with warfarin therapy and adverse clinical outcomes among different ethnic groups across the globe. Findings suggest higher prevalence of AF but better anticoagulation control among the Whites as compared to other ethnicities. Unfortunately, non-whites had higher risk of strokes and bleeding outcomes while on warfarin therapy. Addressing disparities in prevention and healthcare resource allocation will likely improve AF-related outcomes in minorities.

## Author Contributions

HZ: conceptualization and supervision. NZ, IA, and HZ: methodology, formal analysis, investigation, data curation, writing—original draft preparation, and project administration. NZ, IA, LM, and HZ: software and validation. LM, HPG, and HZ: resources, writing—review and editing, and funding acquisition. All authors have read and agreed to the published version of the manuscript.

## Conflict of Interest

The authors declare that the research was conducted in the absence of any commercial or financial relationships that could be construed as a potential conflict of interest.

## Publisher's Note

All claims expressed in this article are solely those of the authors and do not necessarily represent those of their affiliated organizations, or those of the publisher, the editors and the reviewers. Any product that may be evaluated in this article, or claim that may be made by its manufacturer, is not guaranteed or endorsed by the publisher.
